# Temporal trends in normal weight central obesity and its associations with cardiometabolic risk among Chinese adults

**DOI:** 10.1038/s41598-019-41986-5

**Published:** 2019-04-01

**Authors:** Peige Song, Xue Li, Yongjun Bu, Shibin Ding, Desheng Zhai, Erhui Wang, Zengli Yu

**Affiliations:** 1 School of Public Health, Xinxiang Medical University, Xinxiang, China; 2 Centre for Global Health Research, University of Edinburgh, Edinburgh, United Kingdom

## Abstract

Normal weight central obesity (NWCO), a distinct phenotype of obesity that is associated with a higher risk of cardiometabolic dysregulation, has received growing attention in the scientific literature. In this study, we aimed to report the prevalence of NWCO in the general Chinese adults and its secular trend from 1993 to 2011. The comorbid cardiometabolic risk of NWCO was also explored. Data from the China Health and Nutrition Survey (CHNS) 1993–2011 were obtained. NWCO was defined as the combination of a BMI of 18.5–23.9 kg/m^2^ and 1) a waist circumference (WC) of >85 cm in males or >80 cm in females (NWCO by WC); 2) a waist to height ratio (WHtR) of ≥0.5 (NWCO by WHtR); 3) a waist to hip ratio (WHR) of ≥0.9 in males or ≥0.85 in females (NWCO by WHR). We assessed the trend of NWCO prevalence with the generalized estimating equation method. The demographic, socioeconomic, geographic, behavioural and cardiometabolic predictors of NWCO were explored with multivariable logistic regression. From 1993 to 2011, the age-standardized prevalence of NWCO by WC increased from 6.65% (95% CI: 6.09–7.26) to 13.24% (95% CI: 12.58–13.93), and that of NWCO by WHtR and NWCO by WHR rose from 13.18% (95% CI: 12.41–13.98) to 17.06% (95% CI: 16.35–17.79) and from 16.14% (95% CI: 15.3–17.01) to 19.04% (95% CI: 18.25–19.85) respectively. The associated cardiometabolic factors of NWCO (by WC, WHtR and WHR) were hypertension, diabetes, insulin resistance, decreased insulin sensitivity, low high-density lipoprotein and elevated triglyceride. Moreover, NWCO by WC and NWCO by WHtR were associated with a decreased risk of impaired insulin secretion, and NWCO by WC was additionally linked to elevated total cholesterol. The prevalence of NWCO in the general Chinese adults increased significantly from 1993 to 2011. Effective strategies are needed to combat this epidemic and reduce its deleterious health outcomes.

## Introduction

Obesity, defined as a chronic metabolic disorder characterized as an excessive accumulation and storage of body fat, is a well- recognised risk factor for cardiovascular diseases, diabetes and several types of cancer^1,2^. In the general population, obesity is associated with an increased risk of premature death and reduced life expectancy^3,4^. During the last few decades, the prevalence of obesity has remarkably increased in both developed and developing regions, the pandemic of obesity and its devastating threat to health have placed a considerable public health burden on the human society^2,5–7^.

The gold standard for defining obesity is by measuring the excess in body fat^8,9^. However, in epidemiological studies, as well as in routine clinical practices, this approach is not widely used, largely due to the complexity and high expense of necessary techniques and equipment^8^. Body mass index (BMI), defined as weight in kilograms divided by the square of height in meters, has predominantly been adopted as a surrogate of adiposity or obesity^6,7,10,11^. Despite its advantages, such as simplicity and reproducibility, BMI is not optimal in differentiating muscle mass from bone and fat mass, especially in intermediate BMI range^8,9,12^. In addition, the famous “obesity paradoxes”, such as an inverse association between BMI and mortality in people with coronary artery disease (CAD) or heart failure, also challenge the conventional definition of obesity by BMI alone^13–15^. It has been suggested that central obesity (or abdominal obesity), generally assessed by anthropometric measurements such as waist circumference (WC), waist to height ratio (WHtR) and waist to hip ratio (WHR), more accurately describes the distribution of body fat and better predicts obesity-related health risk than general obesity by BMI^16–19^. By combining general obesity and central obesity, a distinct phenotype of obesity-normal weight central obesity (NWCO) has been consequently described and received growing attention^20–22^. Previous studies have suggested a positive association between NWCO and cardiometabolic dysregulation, systemic inflammation, and mortality^21,22^. CAD patients with NWCO also conferred the highest risk of mortality^23,24^.

Despite the growing concerns about NWCO as a risk factor for human health, the prevalence of NWCO has been seldom studied in the general population. In the largest developing country-China, rapid socioeconomic development, nutrition transition towards high-fat and high-energy-density diets and pandemic of physical inactivity have brought obesity an increasing public health challenge^11,25^. In the last three decades, the prevalence of general obesity defined by BMI and that of abdominal obesity defined by WC in Chinese adults were both suggested to have more than doubled^11^. In addition, the prevalence of central obesity (defined by WC) in people with normal weight has increased from 11.9% in 1993 to 21.1% in 2009 in China^26^. In this estimation, NWCO was defined as the combination of a normal BMI and an elevated WC and the target sample was people with normal weight. According to previous evidence, WHtR appears to be a more optimal predictor of cardiometabolic risk and mortality compared with WC, WHR and BMI^10,19,27,28^. Thus far, the prevalence of NWCO in the general Chinese population has never been assessed and revealed across a wide time range, let alone by using multiple definitions of NWCO simultaneously (i.e., combinations of BMI and WHtR, BMI and WHtR, BMI and WHR). Moreover, the association between NWCO and cardiometabolic risk has never been extensively studied in the general Chinese adults.

To fill the research gaps outlined above, in this study, we aimed 1) to present the prevalence of NWCO in the general Chinese adults from 1993 to 2011, by using multiple definitions of NWCO (normal BMI with elevated WC, elevated WHtR and elevated WHR respectively); 2) to explore the variations of NWCO prevalence in different demographic, socioeconomic, geographic, behavioural groups; and 3) to identify the associated cardiometabolic risk of NWCO.

## Results

### Characteristics of participants

From the China Health and Nutrition Survey (CHNS) 1993 to CHNS 2011, a total of 100840 records of adult subjects (excluding pregnant women) were available, of which 61253 records of 22398 unique adults were with complete information on anthropometry (weight, height, WC, hip circumference), demography (age, sex), socioeconomic status (marriage, education, economic level), geography (setting, region) and health-behaviours (smoking, drinking), and thus were included in our analyses (see Fig. S1 for details on the record selection process). The demographic characteristics of included and excluded participants in the analyses are shown in Table S1. Compared with those excluded, the included subjects were relatively older (all p values < 0.001). Sex distribution was broadly similar between the included and excluded groups, except in CHNS 1993 and CHNS 2004, where larger proportions of female participants were in the included groups than in the excluded groups (p = 0.005 in 1993 and p < 0.001 in 2004). The basic characteristics of the 61253 included records are shown in Table S2. For the assessment of cardiometabolic risk, the records of people with normal weight in CHNS 2009 (n = 4244) was selected, the basic characteristics of the 4244 included records are shown in Table S3.

### Trends in the prevalence of normal weight central obesity

**Figure 1:**
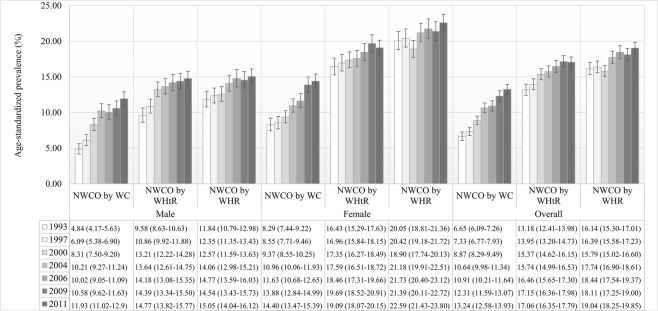
Sex-specific age-standardized prevalence of normal weight central obesity in the general Chinese adults, CHNS 1993–20011.

From 1993 to 2011, the age-standardized prevalence of NWCO by WC increased from 6.65% (95% CI: 6.09–7.26) to 13.24% (95% CI: 12.58–13.93). Within the same time frame, the prevalence of NWCO by WC was generally higher in females than in males, except in 2000–2006, when overlaps of 95% CI occurred. From 1990 to 2011, the age-standardized prevalence of NWCO by WC increased from 4.84% (95% CI: 4.17–5.63) to 11.93% (95% CI: 11.02–12.90) in males, and from 8.29% (95% CI: 7.44–9.22) to 14.40% (95% CI: 13.47–15.39) in females, yielding relative increasing rates of 146.49% (in males) and 73.70% (in females) respectively (Fig. 1). After adjusting for age, the prevalence of NWCO by WC across all demographic, socioeconomic, geographic and behavioural groups increased dramatically from 1993 to 2011 (Table 1).

**Table 1:** Prevalence of normal weight central obesity defined by waist circumference, CHNS 1993–2011.

Characteristic	1993	1997	2000	2004	2006	2009	2011	p for age-adjusted trend	Trend direction
Crude	6.46 (5.92–7.04)	7.38 (6.82–7.99)	9.31 (8.70–9.95)	11.50 (10.84–12.20)	12.16 (11.48–12.88)	13.93 (13.22–14.66)	14.59 (13.97–15.23)	<0.001	Increasing
Age group									
18–29 years	2.95 (2.28–3.82)	4.26 (3.41–5.32)	4.04 (3.15–5.19)	8.21 (6.70–10.02)	7.32 (5.74–9.30)	8.76 (7.06–10.82)	9.56 (7.99–11.40)	<0.001	Increasing
30–39 years	5.83 (4.82–7.02)	4.90 (3.96–6.05)	7.24 (6.17–8.49)	7.11 (5.97–8.45)	8.39 (7.09–9.90)	10.33 (8.85–12.03)	12.37 (10.88–14.02)	<0.001	Increasing
40–49 years	6.06 (4.97–7.36)	8.74 (7.52–10.13)	9.96 (8.72–11.36)	10.76 (9.46–12.21)	11.59 (10.23–13.10)	11.03 (9.74–12.46)	13.57 (12.35–14.89)	<0.001	Increasing
50–59 years	7.79 (6.28–9.62)	9.10 (7.57–10.90)	11.76 (10.17–13.56)	13.86 (12.38–15.49)	12.77 (11.38–14.30)	15.22 (13.76–16.80)	13.86 (12.65–15.17)	<0.001	Increasing
60–69 years	11.86 (9.76–14.35)	10.19 (8.33–12.40)	12.99 (10.96–15.33)	14.15 (12.26–16.28)	14.90 (13.02–16.99)	17.68 (15.80–19.74)	16.47 (14.97–18.08)	<0.001	Increasing
≥70 years	14.53 (11.24–18.57)	13.28 (10.49–16.67)	15.73 (12.93–19.01)	17.38 (14.90–20.16)	19.38 (16.88–22.15)	21.03 (18.65–23.62)	22.05 (19.95–24.30)	<0.001	Increasing
Sex									
Male	4.76 (4.11–5.52)	6.12 (5.40–6.93)	8.59 (7.76–9.50)	10.50 (9.60–11.49)	10.67 (9.74–11.67)	11.63 (10.70–12.62)	13.05 (12.20–13.94)	<0.001	Increasing
Female	8.02 (7.20–8.93)	8.62 (7.78–9.55)	9.99 (9.12–10.93)	12.42 (11.48–13.42)	13.50 (12.52–14.53)	16.02 (14.99–17.09)	15.97 (15.09–16.88)	<0.001	Increasing
Marital status									
Single	5.75 (4.70–7.02)	6.48 (5.37–7.81)	7.80 (6.57–9.25)	12.49 (10.90–14.28)	12.69 (11.00–14.60)	13.88 (12.17–15.79)	14.50 (12.97–16.17)	<0.001	Increasing
Married	6.65 (6.03–7.32)	7.62 (6.98–8.31)	9.66 (8.97–10.39)	11.30 (10.58–12.06)	12.07 (11.33–12.85)	13.93 (13.17–14.73)	14.61 (13.94–15.30)	<0.001	Increasing
Education									
No formal education	8.85 (7.77–10.06)	9.76 (8.59–11.07)	12.85 (11.41–14.45)	15.55 (13.95–17.30)	14.98 (13.51–16.58)	17.88 (16.28–19.60)	18.49 (16.99–20.10)	<0.001	Increasing
Primary education	5.77 (4.72–7.04)	7.63 (6.46–8.98)	7.48 (6.36–8.77)	10.44 (9.17–11.86)	11.93 (10.40–13.66)	14.36 (12.79–16.09)	15.13 (13.62–16.78)	<0.001	Increasing
Middle education	4.39 (3.72–5.18)	5.76 (5.01–6.62)	8.51 (7.66–9.45)	10.32 (9.39–11.32)	10.81 (9.85–11.85)	12.55 (11.56–13.60)	13.23 (12.35–14.17)	<0.001	Increasing
Higher education	11.24 (8.29–15.08)	6.58 (4.73–9.07)	8.96 (7.14–11.20)	10.87 (9.00–13.06)	11.74 (9.95–13.81)	11.03 (9.34–12.99)	13.25 (11.96–14.67)	0.003	Increasing
Economic status									
Poor	6.85 (5.88–7.97)	7.89 (6.88–9.02)	9.35 (8.28–10.53)	13.01 (11.76–14.37)	13.16 (11.86–14.59)	13.38 (12.12–14.75)	14.91 (13.72–16.18)	<0.001	Increasing
Middle	6.44 (5.53–7.49)	6.83 (5.91–7.88)	8.63 (7.63–9.75)	10.70 (9.62–11.90)	11.17 (10.05–12.39)	13.61 (12.42–14.89)	14.76 (13.71–15.88)	<0.001	Increasing
Rich	6.13 (5.27–7.11)	7.46 (6.53–8.51)	9.89 (8.86–11.02)	11.00 (9.95–12.15)	12.29 (11.21–13.47)	14.62 (13.47–15.85)	14.23 (13.27–15.25)	<0.001	Increasing
Setting									
Rural	5.72 (5.11–6.40)	6.68 (6.03–7.38)	8.45 (7.75–9.20)	10.88 (10.11–11.70)	11.67 (10.87–12.53)	13.45 (12.62–14.32)	14.09 (13.29–14.92)	<0.001	Increasing
Urban	8.03 (6.99–9.20)	8.90 (7.84–10.10)	11.15 (10.00–12.41)	12.89 (11.67–14.23)	13.26 (12.01–14.62)	14.98 (13.71–16.36)	15.27 (14.31–16.28)	<0.001	Increasing
Region									
North	8.97 (7.91–10.14)	9.30 (8.27–10.44)	10.98 (9.97–12.07)	11.73 (10.73–12.81)	12.98 (11.93–14.12)	14.60 (13.53–15.74)	13.61 (12.68–14.60)	<0.001	Increasing
South	5.13 (4.54–5.79)	6.30 (5.66–7.02)	8.12 (7.38–8.92)	11.33 (10.47–12.26)	11.54 (10.66–12.48)	13.40 (12.49–14.37)	15.24 (14.44–16.09)	<0.001	Increasing
Smoking									
Non-smoker	7.18 (6.48–7.94)	7.69 (6.99–8.45)	9.38 (8.65–10.17)	11.61 (10.81–12.47)	12.63 (11.79–13.51)	14.74 (13.87–15.64)	15.19 (14.44–15.98)	<0.001	Increasing
Smoker	5.07 (4.27–6.00)	6.76 (5.84–7.81)	9.15 (8.10–10.31)	11.28 (10.16–12.51)	11.16 (10.02–12.42)	12.16 (11.01–13.42)	13.23 (12.18–14.36)	<0.001	Increasing
Alcohol drinking									
Non-drinker	7.06 (6.36–7.83)	7.55 (6.84–8.33)	9.51 (8.75–10.33)	11.68 (10.88–12.54)	12.80 (11.96–13.69)	15.01 (14.12–15.94)	15.15 (14.38–15.96)	<0.001	Increasing
Drinker	5.40 (4.60–6.33)	7.09 (6.20–8.10)	8.94 (7.96–10.03)	11.14 (10.03–12.36)	10.82 (9.70–12.05)	11.76 (10.65–12.96)	13.50 (12.49–14.57)	<0.001	Increasing

Note: values were prevalence estimates (95% confidence interval); *age-adjusted trends in the prevalence of NWO from 1993 to 2011 were assessed by the generalized-estimating-equation method.

For NWCO by WHtR, the age-standardized prevalence also increased from 13.18% (95% CI: 12.41–13.98) to 17.06% (95% CI: 16.35–17.79) during 1993–2011. NWCO by WHtR was consistently more prevalent in females than in males. From 1993 to 2011, the prevalence of NWCO by WHtR rose from 9.58% (95% CI: 8.63–10.63) to 14.77% (95% CI: 13.82–15.77) in males and 16.43% (95% CI: 15.29–17.63) to 19.09% (95% CI: 18.07–20.15) in females, with the relative increasing rate in males being more than triple of that in females (54.18% vs. 16.19%) (Fig. 1). The linear increasing trend of NWCO by WHtR prevalence was statistically significant in nearly all demographic, socioeconomic, geographic and behavioural groups from 1993 to 2011, except in people aged 40–49 years, people with higher education and people residing in North China (Table 2).

**Table 2:** Prevalence of normal weight central obesity defined by waist to height ratio, CHNS 1993–2011.

Characteristic	1993	1997	2000	2004	2006	2009	2011	p for age-adjusted trend	Trend direction
Crude	12.61 (11.87–13.39)	14.09 (13.33–14.89)	16.09 (15.32–16.91)	17.78 (16.98–18.60)	19.16 (18.33–20.01)	20.51 (19.69–21.36)	19.96 (19.26–20.68)	<0.001	Increasing
Age group									
18–29 years	5.22 (4.30–6.31)	6.39 (5.33–7.65)	7.20 (5.98–8.64)	8.21 (6.70–10.02)	9.12 (7.35–11.28)	8.99 (7.27–11.07)	9.64 (8.07–11.49)	<0.001	Increasing
30–39 years	9.54 (8.25–11.01)	9.92 (8.58–11.45)	11.36 (10.02–12.86)	11.69 (10.23–13.33)	11.62 (10.10–13.34)	12.95 (11.30–14.81)	15.43 (13.79–17.23)	<0.001	Increasing
40–49 years	13.85 (12.22–15.66)	16.10 (14.47–17.86)	16.84 (15.25–18.55)	16.65 (15.06–18.37)	16.53 (14.93–18.26)	16.67 (15.11–18.35)	16.39 (15.07–17.81)	0.085	No trend
50–59 years	17.28 (15.06–19.75)	18.46 (16.32–20.80)	20.65 (18.60–22.85)	21.19 (19.41–23.09)	21.81 (20.06–23.67)	22.66 (20.94–24.48)	19.85 (18.44–21.34)	0.028	Increasing
60–69 years	20.99 (18.25–24.02)	20.61 (18.03–23.46)	24.45 (21.78–27.35)	24.25 (21.86–26.80)	25.68 (23.33–28.19)	27.74 (25.49–30.11)	25.30 (23.52–27.16)	<0.001	Increasing
≥70 years	30.45 (25.89–35.42)	27.19 (23.35–31.42)	28.03 (24.44–31.92)	28.38 (25.36–31.60)	32.30 (29.26–35.48)	33.72 (30.90–36.66)	33.00 (30.58–35.51)	0.019	Increasing
Sex									
Male	9.16 (8.25–10.16)	10.87 (9.92–11.90)	13.76 (12.73–14.86)	14.91 (13.85–16.04)	16.11 (15.00–17.29)	17.18 (16.08–18.34)	17.53 (16.57–18.54)	<0.001	Increasing
Female	15.81 (14.69–17.01)	17.25 (16.09–18.47)	18.31 (17.17–19.51)	20.39 (19.24–21.60)	21.86 (20.67–23.10)	23.54 (22.35–24.78)	22.13 (21.13–23.16)	<0.001	Increasing
Marital status									
Single	11.12 (9.65–12.77)	12.52 (10.98–14.25)	14.18 (12.53–16.01)	18.74 (16.82–20.81)	21.50 (19.37–23.81)	21.32 (19.26–23.53)	21.21 (19.42–23.13)	<0.001	Increasing
Married	13.02 (12.17–13.91)	14.50 (13.64–15.40)	16.54 (15.67–17.45)	17.58 (16.71–18.48)	18.72 (17.83–19.65)	20.36 (19.47–21.29)	19.73 (18.98–20.51)	<0.001	Increasing
Education									
No formal education	19.30 (17.76–20.93)	21.60 (19.93–23.36)	25.12 (23.20–27.14)	27.26 (25.25–29.37)	28.52 (26.61–30.50)	29.56 (27.62–31.57)	29.45 (27.66–31.30)	<0.001	Increasing
Primary education	11.86 (10.35–13.55)	14.67 (13.07–16.42)	14.84 (13.29–16.54)	17.95 (16.32–19.70)	19.08 (17.19–21.13)	21.63 (19.75–23.63)	23.28 (21.47–25.19)	<0.001	Increasing
Middle education	7.69 (6.80–8.70)	9.46 (8.50–10.52)	13.14 (12.09–14.26)	14.38 (13.30–15.52)	15.60 (14.46–16.80)	17.32 (16.19–18.52)	16.89 (15.90–17.91)	<0.001	Increasing
Higher education	13.02 (9.83–17.05)	9.09 (6.90–11.89)	11.56 (9.48–14.02)	13.06 (11.02–15.41)	13.51 (11.59–15.69)	13.86 (11.96–15.99)	14.58 (13.23–16.05)	0.073	No trend
Economic status									
Poor	15.07 (13.65–16.60)	15.81 (14.41–17.32)	17.15 (15.74–18.65)	20.35 (18.84–21.96)	21.77 (20.16–23.48)	21.40 (19.87–23.03)	22.56 (21.15–24.03)	<0.001	Increasing
Middle	12.06 (10.83–13.42)	13.42 (12.15–14.80)	15.85 (14.52–17.28)	17.06 (15.72–18.49)	18.90 (17.49–20.39)	19.82 (18.42–21.29)	20.01 (18.81–21.26)	<0.001	Increasing
Rich	11.00 (9.86–12.26)	13.18 (11.96–14.51)	15.40 (14.14–16.75)	16.33 (15.07–17.66)	17.47 (16.20–18.82)	20.44 (19.12–21.83)	18.18 (17.11–19.29)	<0.001	Increasing
Setting									
Rural	12.10 (11.22–13.03)	14.19 (13.27–15.16)	16.14 (15.2–17.13)	18.23 (17.27–19.24)	19.65 (18.65–20.69)	20.61 (19.62–21.64)	21.22 (20.28–22.20)	<0.001	Increasing
Urban	13.71 (12.37–15.16)	13.88 (12.57–15.31)	15.99 (14.64–17.45)	16.76 (15.38–18.24)	18.06 (16.63–19.59)	20.30 (18.85–21.84)	18.24 (17.21–19.32)	<0.001	Increasing
Region									
North	13.98 (12.68–15.39)	13.15 (11.95–14.46)	15.34 (14.17–16.59)	15.13 (14.01–16.33)	16.99 (15.81–18.25)	17.80 (16.63–19.03)	15.85 (14.85–16.90)	0.944	No trend
South	11.89 (11.00–12.83)	14.62 (13.66–15.64)	16.63 (15.61–17.71)	19.78 (18.68–20.93)	20.82 (19.69–22.00)	22.62 (21.49–23.80)	22.72 (21.77–23.69)	<0.001	Increasing
Smoking									
Non-smoker	14.17 (13.21–15.18)	15.03 (14.08–16.03)	16.71 (15.75–17.71)	18.51 (17.53–19.54)	20.09 (19.07–21.14)	21.50 (20.49–22.55)	20.55 (19.70–21.42)	<0.001	Increasing
Smoker	9.61 (8.52–10.83)	12.17 (10.96–13.51)	14.79 (13.48–16.20)	16.27 (14.95–17.69)	17.14 (15.75–18.62)	18.37 (16.98–19.84)	18.64 (17.42–19.92)	<0.001	Increasing
Alcohol drinking									
Non-drinker	13.71 (12.75–14.73)	15.11 (14.13–16.14)	17.03 (16.04–18.07)	19.09 (18.09–20.13)	20.53 (19.50–21.60)	22.11 (21.08–23.18)	21.26 (20.37–22.17)	<0.001	Increasing
Drinker	10.69 (9.57–11.92)	12.35 (11.19–13.61)	14.41 (13.19–15.73)	15.12 (13.84–16.49)	16.27 (14.93–17.71)	17.32 (16.00–18.72)	17.46 (16.33–18.65)	<0.001	Increasing

Note: values were prevalence estimates (95% confidence interval); *age-adjusted trends in the prevalence of NWO from 1993 to 2011 were assessed by the generalized-estimating-equation method.

For NWCO by WHR, the age-standardized prevalence rose from 16.14% (95% CI: 15.3–17.01) to 19.04% (95% CI: 18.25–19.85) during 1993–2011. Similar to NWCO by WHtR, the age-standardized prevalence of NWCO by WHR was consistently lower in males than in females. Within this time frame, the age-standardized prevalence of NWCO by WHR ranged from 11.84% (95% CI: 10.79–12.98) to 15.05% (95% CI: 14.04–16.12) in males, and from 20.05% (95% CI: 18.81–21.36) to 22.59% (95% CI: 21.43–23.80) in females during 1993–2011. The corresponding relative increasing rate in males was greater than that in females (27.11% vs. 12.67%) (Fig. 1). However, no statistically significant secular trend of increasing NWCO by WHR prevalence was observed among people aged 50–59 years or ≥70 years, single people, people with no formal education, and people in poor or middle economic status from 1993 to 2011 (Table 3).

**Table 3:** Prevalence of normal weight central obesity defined by waist to hip ratio, CHNS 1993–2011.

Characteristic	1993	1997	2000	2004	2006	2009	2011	p for age-adjusted trend	Trend direction
Crude	15.63 (14.81–16.48)	16.37 (15.56–17.21)	16.19 (15.41–17.00)	18.66 (17.85–19.50)	19.73 (18.89–20.60)	20.22 (19.40–21.07)	20.35 (19.64–21.07)	<0.001	Increasing
Age group									
18–29 years	11.22 (9.88–12.72)	12.73 (11.24–14.38)	11.10 (9.59–12.82)	14.81 (12.80–17.08)	15.37 (13.07–17.98)	13.77 (11.64–16.21)	16.16 (14.14–18.40)	<0.001	Increasing
30–39 years	12.45 (10.98–14.08)	11.89 (10.43–13.54)	14.59 (13.08–16.24)	15.67 (14.00–17.50)	15.65 (13.91–17.57)	14.37 (12.63–16.29)	18.02 (16.26–19.92)	<0.001	Increasing
40–49 years	15.53 (13.81–17.42)	17.15 (15.48–18.95)	15.27 (13.75–16.92)	16.70 (15.11–18.42)	17.41 (15.78–19.17)	17.16 (15.58–18.86)	17.89 (16.52–19.36)	0.042	Increasing
50–59 years	20.48 (18.09–23.09)	20.28 (18.06–22.70)	17.92 (16.00–20.02)	20.35 (18.60–22.22)	20.17 (18.47–21.98)	21.36 (19.68–23.14)	18.95 (17.57–20.42)	0.879	No trend
60–69 years	20.73 (18.01–23.75)	19.91 (17.36–22.72)	21.72 (19.17–24.52)	21.92 (19.63–24.39)	23.35 (21.08–25.79)	23.99 (21.86–26.27)	22.64 (20.94–24.45)	0.022	Increasing
≥70 years	30.45 (25.89–35.42)	26.77 (22.94–30.97)	24.95 (21.52–28.74)	26.00 (23.08–29.15)	29.99 (27.03–33.13)	32.17 (29.39–35.09)	30.85 (28.48–33.33)	0.210	No trend
Sex									
Male	11.49 (10.47–12.58)	12.32 (11.31–13.4)	12.96 (11.96–14.04)	14.71 (13.65–15.83)	15.45 (14.36–16.62)	16.22 (15.15–17.36)	16.54 (15.60–17.52)	<0.001	Increasing
Female	19.47 (18.24–20.76)	20.33 (19.10–21.63)	19.26 (18.10–20.48)	22.25 (21.06–23.50)	23.53 (22.30–24.80)	23.86 (22.67–25.11)	23.75 (22.72–24.81)	0.001	Increasing
Marital status									
Single	16.74 (14.97–18.67)	15.26 (13.56–17.12)	15.86 (14.13–17.77)	20.03 (18.06–22.15)	22.80 (20.61–25.14)	21.46 (19.39–23.68)	22.82 (20.97–24.79)	0.085	No trend
Married	15.32 (14.42–16.28)	16.65 (15.74–17.61)	16.27 (15.40–17.17)	18.37 (17.49–19.29)	19.16 (18.26–20.09)	19.99 (19.10–20.91)	19.90 (19.14–20.68)	<0.001	Increasing
Education									
No formal education	22.27 (20.65–23.99)	23.18 (21.47–24.99)	23.09 (21.23–25.05)	25.08 (23.13–27.14)	26.06 (24.22–27.99)	27.75 (25.85–29.73)	27.95 (26.19–29.78)	0.050	No trend
Primary education	14.01 (12.39–15.82)	16.59 (14.90–18.42)	15.44 (13.86–17.16)	18.91 (17.25–20.70)	17.90 (16.06–19.91)	20.53 (18.69–22.50)	21.42 (19.67–23.28)	<0.001	Increasing
Middle education	11.33 (10.25–12.51)	12.45 (11.36–13.64)	14.05 (12.97–15.20)	16.07 (14.94–17.27)	17.40 (16.21–18.65)	17.42 (16.28–18.62)	17.58 (16.58–18.62)	<0.001	Increasing
Higher education	14.79 (11.39–19.00)	11.03 (8.60–14.03)	11.69 (9.60–14.16)	16.36 (14.09–18.90)	18.17 (15.98–20.60)	16.15 (14.12–18.41)	18.03 (16.55–19.62)	<0.001	Increasing
Economic status									
Poor	17.94 (16.41–19.58)	18.32 (16.83–19.90)	18.00 (16.56–19.53)	20.23 (18.72–21.84)	21.43 (19.83–23.13)	21.44 (19.90–23.06)	22.25 (20.85–23.72)	0.059	No trend
Middle	16.29 (14.88–17.81)	16.76 (15.36–18.26)	15.52 (14.2–16.93)	18.09 (16.71–19.55)	19.08 (17.66–20.58)	19.31 (17.93–20.77)	20.06 (18.86–21.31)	0.085	No trend
Rich	13.02 (11.78–14.36)	14.25 (12.98–15.62)	15.23 (13.97–16.57)	17.89 (16.59–19.27)	19.05 (17.73–20.44)	20.09 (18.78–21.47)	19.32 (18.23–20.46)	<0.001	Increasing
Setting									
Rural	16.19 (15.19–17.24)	17.80 (16.78–18.85)	16.96 (16.00–17.97)	18.86 (17.88–19.88)	19.53 (18.53–20.57)	20.45 (19.46–21.47)	20.81 (19.87–21.78)	0.016	Increasing
Urban	14.43 (13.07–15.91)	13.31 (12.02–14.72)	14.54 (13.24–15.94)	18.20 (16.78–19.72)	20.18 (18.68–21.76)	19.73 (18.29–21.25)	19.72 (18.66–20.83)	<0.001	Increasing
Region									
North	14.97 (13.63–16.41)	14.31 (13.05–15.66)	14.81 (13.66–16.04)	16.14 (14.99–17.37)	18.06 (16.85–19.35)	18.38 (17.2–19.63)	17.24 (16.21–18.33)	0.003	Increasing
South	15.97 (14.96–17.04)	17.53 (16.49–18.62)	17.17 (16.13–18.27)	20.56 (19.45–21.73)	21.01 (19.88–22.19)	21.65 (20.53–22.81)	22.43 (21.48–23.40)	<0.001	Increasing
Smoking									
Non-smoker	17.43 (16.38–18.52)	17.71 (16.69–18.77)	17.28 (16.31–18.29)	19.61 (18.60–20.65)	21.13 (20.09–22.21)	21.47 (20.46–22.51)	21.59 (20.73–22.49)	<0.001	Increasing
Smoker	12.17 (10.94–13.50)	13.64 (12.35–15.03)	13.87 (12.60–15.25)	16.70 (15.36–18.13)	16.69 (15.31–18.16)	17.52 (16.16–18.97)	17.54 (16.35–18.80)	<0.001	Increasing
Alcohol drinking									
Non-drinker	17.25 (16.19–18.36)	17.68 (16.63–18.78)	17.64 (16.63–18.69)	20.09 (19.07–21.15)	21.14 (20.1–22.22)	21.94 (20.91–23.01)	21.77 (20.88–22.69)	<0.001	Increasing
Drinker	12.79 (11.58–14.11)	14.11 (12.88–15.45)	13.60 (12.40–14.88)	15.76 (14.46–17.15)	16.75 (15.39–18.21)	16.78 (15.49–18.17)	17.60 (16.47–18.79)	<0.001	Increasing

Note: values were prevalence estimates (95% confidence interval); *age-adjusted trends in the prevalence of NWO from 1993 to 2011 were assessed by the generalized-estimating-equation method.

### Demographic, socioeconomic, geographic and behavioural predictors of normal weight central obesity

Both univariable and multivariable logistic regression GEE models were performed to investigate the potential demographic, socioeconomic, geographic and behavioural predictors of NWCO (Tables S4 and 4). As revealed in multivariable logistic regression GEE models (Table 4), NWCO was strongly associated with the survey year, where the prevalence rates of NWCO by WC and NWCO by WHtR increased gradually with survey years after adjusting for demographic characteristics, socioeconomic status, geographic location and health behaviours. For NWCO by WHR, it was progressive more common in 2004, 2006, 2009 and 2011 than in 1993 (Table 4). Moreover, advanced age, female sex, being married, and urban residence (vs. rural residence) were all positive predictors of NWCO by WC, whereas people with primary or middle education were less likely to have NWCO by WC compared with those with no formal education. The relative risk of NWCO by WC was also lower among people in South China than among those in North China. For NWCO by WHtR, advanced age, female sex, and living in South China (vs. in North China) were all positively associated with higher odds. However, higher educational attainment (vs. no formal education), higher economic level (vs. poor level) and urban residence (vs. rural residence) were associated with a decreased risk of NWCO by WHtR. For NWCO by WHR, advanced age was also a positive predictor, where people aged 40 years and above had a higher risk of NWCO by WHR than those aged 18–29 years. Moreover, female sex and residing in South China were also negative predictors of NWCO by WHR. A consistent inverse association existed between higher education attainments or economic levels and the prevalence of NWCO by WHR. People living in urban areas had an elevated risk of NWCO by WHR compared with those in rural areas. Neither smoking nor alcohol drinking was independently associated with higher risk of NWCO by WC, by WHtR and by WHR.

**Table 4:** Adjusted odds ratios of demographic, socioeconomic and behavioural factors of normal weight central obesity in multivariable logistic regressions, CHNS 1993–2011.

Characteristic	NWCO by WC		NWCO by WHtR		NWCO by WHR	
	Adjusted odds ratio (95% CI)	p value	Adjusted odds ratio (95% CI)	p value	Adjusted odds ratio (95% CI)	p value
Survey year		<**0**.**001**		<**0**.**001**		<**0**.**001**
1993	1.00 (reference)		1.00 (reference)		1.00 (reference)	
1997	1.13 (1.00–1.27)	0.046	1.11 (1.01–1.21)	0.024	1.06 (0.97–1.15)	0.19
2000	1.42 (1.26–1.60)	<0.001	1.29 (1.18–1.41)	<0.001	1.05 (0.97–1.14)	0.256
2004	1.69 (1.51–1.89)	<0.001	1.34 (1.23–1.47)	<0.001	1.18 (1.09–1.29)	<0.001
2006	1.76 (1.57–1.97)	<0.001	1.42 (1.30–1.56)	<0.001	1.25 (1.15–1.36)	<0.001
2009	2.02 (1.81–2.26)	<0.001	1.52 (1.39–1.66)	<0.001	1.27 (1.17–1.38)	<0.001
2011	2.09 (1.87–2.33)	<0.001	1.48 (1.36–1.62)	<0.001	1.28 (1.18–1.39)	<0.001
Age group		<**0**.**001**		<**0**.**001**		<**0**.**001**
18–29 years	1.00 (reference)		1.00 (reference)		1.00 (reference)	
30–39 years	1.24 (1.10–1.40)	<0.001	1.53 (1.38–1.70)	<0.001	1.07 (0.98–1.16)	0.154
40–49 years	1.62 (1.44–1.82)	<0.001	2.13 (1.92–2.36)	<0.001	1.22 (1.12–1.34)	<0.001
50–59 years	1.88 (1.66–2.13)	<0.001	2.64 (2.38–2.94)	<0.001	1.43 (1.31–1.57)	<0.001
60–69 years	2.21 (1.95–2.52)	<0.001	3.24 (2.90–3.62)	<0.001	1.60 (1.45–1.77)	<0.001
≥70 years	2.87 (2.49–3.30)	<0.001	4.25 (3.77–4.80)	<0.001	2.20 (1.97–2.45)	<0.001
Sex						
Male	1.00 (reference)		1.00 (reference)		1.00 (reference)	
Female	1.32 (1.22–1.43)	<0.001	1.44 (1.34–1.54)	<0.001	1.67 (1.56–1.79)	<0.001
Marital status						
Single	1.00 (reference)		1.00 (reference)		1.00 (reference)	
Married	1.10 (1.01–1.20)	0.033	1.06 (0.99–1.14)	0.119	1.03 (0.96–1.10)	0.416
Education		**0.071**		<**0**.**001**		**0.001**
No formal education	1.00 (reference)		1.00 (reference)		1.00 (reference)	
Primary education	0.90 (0.83–0.98)	0.016	0.87 (0.81–0.94)	<0.001	0.87 (0.81–0.93)	<0.001
Middle education	0.91 (0.84–0.99)	0.03	0.80 (0.75–0.86)	<0.001	0.85 (0.79–0.91)	<0.001
Higher education	0.90 (0.80–1.02)	0.086	0.75 (0.67–0.83)	<0.001	0.89 (0.81–0.99)	0.03
Economic status		**0.983**		**0.003**		**0.001**
Poor	1.00 (reference)		1.00 (reference)		1.00 (reference)	
Middle	0.97 (0.91–1.03)	0.3	0.92 (0.88–0.97)	0.004	0.93 (0.88–0.98)	0.004
Rich	1.00 (0.93–1.06)	0.914	0.92 (0.87–0.97)	0.002	0.91 (0.86–0.96)	0.001
Setting						
Rural	1.00 (reference)		1.00 (reference)		1.00 (reference)	
Urban	1.17 (1.09–1.25)	<0.001	0.92 (0.87–0.98)	0.006	0.88 (0.83–0.93)	<0.001
Region						
North	1.00 (reference)		1.00 (reference)		1.00 (reference)	
South	0.85 (0.80–0.90)	<0.001	1.20 (1.14–1.27)	<0.001	1.21 (1.15–1.28)	<0.001
Smoking						
Non-smoker	1.00 (reference)		1.00 (reference)		1.00 (reference)	
Smoker	1.02 (0.95–1.11)	0.542	1.03 (0.97–1.10)	0.351	1.06 (0.99–1.13)	0.08
Alcohol drinking						
Non-drinker	1.00 (reference)		1.00 (reference)		1.00 (reference)	
Drinker	1.04 (0.97–1.12)	0.219	1.01 (0.95–1.07)	0.853	1.02 (0.97–1.08)	0.437

*Note: values were odds ratios (95% confidence interval); NWCO = normal weight central obesity; WC = waist circumference; WHtR = waist to height ratio; WHR = waist to hip ratio; Comparisons were between the NWCO group and the control group (without NWCO); The multivariable generalized estimating equations (GEE) logistic regression adjusted all variables listed.

### Cardiometabolic risk associated with normal weight central obesity

**Figure 2:**
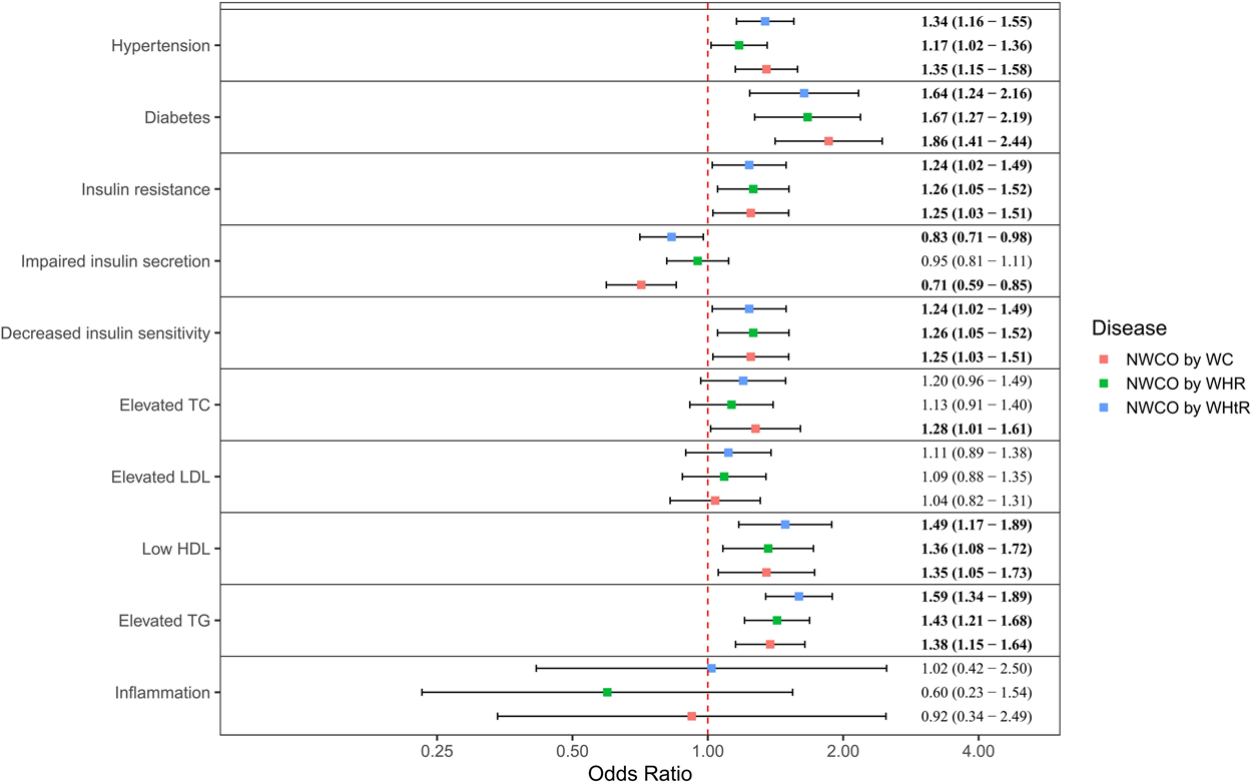
Cardiometabolic risk associated with normal weight central obesity, CHNS 2009. *Note: Demographic, socioeconomic, geographic and behavioural factors were adjusted; Values were odds ratio (95% confidence interval), statistically significant odds ratios are shown in bold; Comparisons were between the NWCO group and the control group (normal weight without central obesity).

As shown in Fig. 2, the cardiometabolic risk associated with NWCO was assessed after adjusting the above-mentioned demographic, socioeconomic, geographic and behavioural factors. In this cross-sectional analysis, people with NWCO (by WC, by WHtR and by WHR) had a higher risk of hypertension (HP), diabetes, insulin resistance (IR), decreased insulin sensitivity (DIS), low high-density lipoprotein (HDL) and elevated triglyceride (TG) compared with people who had normal weight and no central obesity. When taken individually, NWCO by WC and NWCO by WHtR were both associated with a decreased risk of β-cell secretory function (impaired insulin secretion, IIS), and NWCO by WC was additionally linked to the presence of elevated total cholesterol (TC). No significant association between NWCO and elevated low-density lipoprotein (LDL) or inflammation was observed.

## Discussion

In this large longitudinal household-based survey involving a sizeable sample of Chinese adults across the country, the prevalence of NWCO was estimated using three different definitions of NWCO. During 1993–2011, the prevalence of NWCO by WC, by WHtR and by WHR increased dramatically. In 2011, the age-standardized prevalence rates of NWCO by WC, NWCO by WHtR and NWCO by WHR were 13.24% (95% CI: 12.58–13.93), 17.06% (95% CI: 16.35–17.79) and 19.04% (95% CI: 18.25–19.85), translating to an overall 140 million, 180 million and 201 million affected adults respectively in China in 2010. The prevalence of NWCO varied by different definitions, as well as in different demographic, socioeconomic and geographic groups. The cardiometabolic risk factors for NWCO (by WC, WHtR and WHR) were HP, diabetes, IR, DIS, low-HDL and elevated TG. Furthermore, NWCO by WC was additionally associated with a decreased risk of IIS and an increased risk of elevated TC, and NWCO by WHtR was additionally associated with a decreased risk of IIS.

To the best of our knowledge, this is the first study that reported the prevalence of NWCO in the general Chinese adults. The stringent sampling approach and quality-control process largely ensured the reliability of our findings^29,30^. The diagnosis of obesity has been evolving over time^1,2^. In this study, we adopted general obesity by using the widely acknowledged BMI cut-offs for Chinese, and central obesity by using three different indicators, i.e., WC, WHtR and WHR^31–33^. The recently published study by TT Du and colleagues was also based on data from CHNS, however, they specifically aimed to evaluate the central obesity prevalence in people with normal weight^26^. According to their study, where central obesity was defined as a WC ≥ 90 cm in males or ≥80 cm in females, the prevalence of central obesity in people with normal weight (BMI < 25 kg/m^2^) increased from 11.9% to 21.1% during 1993–2009^26^. Despite the same target sample of research and a common increasing secular trend of prevalence in both their study and ours, the current study distinguishes itself by providing the prevalence of NWCO in the general population. Therefore, it is not surprising that our prevalence estimates of NWCO by WC (18.5 kg/m^2^ ≤ BMI < 24.0 kg/m^2^ and WC > 85 cm in males or >80 cm in females) are somewhat lower than those in their study. In Thailand, the prevalence of NWCO, defined as the combination of a BMI below 25 kg/m^2^ and a WHtR of at least 0.5, was revealed to be 15.4% in Thai healthcare providers during 2012 to 2013^34^. In comparison with their results, our study observed a slightly higher age-standardized prevalence of NWCO by WHtR in the general Chinese adults (17.06%) in 2011. Given that the prevalence of NWCO increased with advanced age as reported in the current study, this disparity might be explained by the relatively younger age structure of the include Thai healthcare providers than that of our sample (mean age: 40.20 years vs. 43.47 years).

Irrespective of the adopted definitions, older people and females were revealed to be at a higher risk, but people with higher education level were at a lower risk, of NWCO. From middle age onwards, the volume of subcutaneous fat decreases and fat redistributes from subcutaneous to visceral depots^35^. The sex differences in body composition have long been recognized to be driven by sex steroid hormones, with males having greater lean mass and bone mineral mass, but lower fat mass than females^35^. The inverse association between educational attainment and NWCO has been previously described, whose possible contributors might include higher awareness of obesity and healthier lifestyles in educated people, implying the necessity of promoting obesity-related health education, even in people with normal weight^26,36^.

The odds of NWCO by WC were higher in urban residents than in rural residents, and in North China than in South China^37,38^. Physical inactivity and unhealthy diets that are associated with urbanization can partly explain the urban-rural gradient in NWCO by WC. For the geographic variation of NWCO by WC between North and South China, lifestyles, such as dietary composition, might contribute. Moreover, genetics should also be considered as an important factor for assessing this geographic disparity of NWCO by WC as suggested by a previous study on central obesity^38^. Interestingly, the odds of NWCO by WHtR and NWCO by WHR were both higher among people in rural areas and those in South China, which is exactly in contrast with the pattern of NWCO by WC. According to the definitions, a higher value of WHtR can be the result of either a larger fat mass at the waist or a lower height, and a higher WHR could be resulted by either a greater WC or a lower muscle on the hips. In the sample of the current study, lower values of height and hip circumference were observed in rural and Southern residents (data not presented), therefore resulting in ununiform distributions of NWCO prevalence by different definitions. Both smoking and alcohol drinking were found to be statistically insignificant predictors of NWCO (by WC, by WHtR and by WHR). Although smoking and alcohol have been previously suggested as major risk factors for general obesity and central obesity, it is still not surprising to get such a result given that our target sample were people with NWCO, and the comparison group included underweight, overweight and obese people^39,40^.

The deleterious effects of visceral fat on health have been widely acknowledged^22,31,41^. In this study, NWCO was associated with HP, diabetes, IR, DIS, low-HDL and elevated TG, underscoring the importance of monitoring central obesity in normal-weight people. Previous studies suggested that WC and WHtR were more useful than WHR in identifying cardiovascular risk factors in Chinese subjects^41^. IR and DIS have been marked as features of NWCO in previous studies^22,42^. According to a study in Brazil, where people with NWCO was detected by the combination of a normal BMI (18.5 to 24.9 kg/m^2^) and a higher sum of triceps and subscapular skinfolds (>=90^th^ percentile of the study sample), NWCO was associated with IR and DIS^42^. In our study, we also demonstrated that NWCO by WC and NWCO by WHtR were additionally associated with a decreased risk of IIS, the negative association of NWCO and IIS might be a compensatory result of DIS in people with NWCO^42^. Moreover, NWCO by WC was additionally linked to elevated TC, indicating the superior role of WC and WHtR in assessing cardiometabolic risk among people with normal weight^28^.

However, this study is not free from limitations. First, despite the fact that CHNS is a large-scale survey that represents a geographically wide population of China, its national representativeness cannot be well-guaranteed. Moreover, our prevalence estimates of NWCO were only conducted for North and South China. Those for different regions based on other geographic classifications, such as the East, Central and West regions, were not assessed due to the lack of samples in those areas. Second, our study presented the most comprehensive prevalence estimation of NWCO by using three different definitions, although WC seemed to be superior in assessing cardiometabolic risk with NWCO, the best diagnostic pathway of NWCO cannot be recommended by our study. Third, the cardiometabolic risk of NWCO was assessed by only using the CHNS 2009 database, therefore the statistical power was not as strong as in the assessment of demographic, socioeconomic, geographic and behavioural factors, where pooled data from CHNS 1993–2011 were used.

In conclusion, the prevalence of NWCO in general Chinese adults increased significantly among nearly all demographic, socioeconomic and geographic groups from 1993 to 2011. NWCO was associated with multiple cardiometabolic risk factors. Effective preventive and treatment strategies are urgently needed to combat this epidemic and reduce deleterious obesity-related health outcomes.

## Materials and Methods

### Study Design and study population

Data from CHNS were used in this study. The study design and implement of CHNS have been previously published and detailed elsewhere^29,30^. In brief, CNHS is an ongoing, longitudinal household-based survey covering 56% of Chinese population from 15 provinces (Beijing, Chongqing, Guangxi, Guizhou, Heilongjiang, Henan, Hubei, Hunan, Jiangsu, Liaoning, Shaanxi, Shandong, Shanghai, Yunnan, and Zhejiang) in Mainland China. Until recently, CHNS has been conducted for 10 rounds in 1989, 1991, 1993, 1997, 2000, 2004, 2006, 2009, 2011 and 2015. In every round, samples in each province were drawn through a multistage, random cluster process. First, all counties and cities within each province were stratified by income stratum (low, middle and high), out of which four counties and two cities were randomly selected using a weighted sampling scheme; Second, villages and townships within the selected counties and urban and suburban neighbourhoods within the selected cities were randomly selected, out of which 20 households were randomly chosen; Third, all members of the selected households were interviewed. The protocols of CNHS were approved by the institutional review committees of the University of North Carolina at Chapel Hill, the National Institute of Nutrition and Food Safety, Chinese Center for Disease Control and Prevention, and the China-Japan Friendship Hospital, Ministry of Health. Every participant in CHNS provided written informed consent. All methods were performed in accordance with relevant guidelines and regulations.

For estimating the secular trend of NWCO prevalence and its variations among different demographic, socioeconomic, geographic and behavioural groups, we used data from CHNS 1993, 1997, 2000, 2004, 2006, 2009 and 2011, where WC measurements were available. For exploring the cardiometabolic risk of NWCO, only data from CHNS 2009 were used because of the availability of biomarker data. According to the study context, all participants (except pregnant women) should be aged 18 years or older at the survey. Records with extreme values (the so-called biologically implausible values) for adults (i.e. weight < 20 or >200 kg, height < 40 cm, WC > 200 cm, hip circumstance > 200 cm, BMI > 200 kg/m^2^) were excluded.

### Data collection

Information on demography (e.g. age, sex), socioeconomic stratum (e.g. marital status, educational attainment, income), geographic location (e.g. rural vs. urban, North vs. South), individual lifestyle (e.g. smoking, drinking) and medical history has been obtained by using a structured questionnaire. Anthropometric measurements were carried out following standardized protocols from the World Health Organization (WHO)^11,43^. Weight was measured to the nearest 0.1 kg in light clothing by using a calibrated beam scale, and height was measured to the nearest 0.1 cm without shoes by using a portable stadiometer. With a non-elastic tape, WC was measured to the nearest 0.1 cm at the midpoint between the lowest rib and the iliac crest in a horizontal plane^11,43,44^. BMI was calculated as weight (in kg) divided by the square of height (in m^2^), WHtR as WC (in cm) divided by height (in cm), and WHR as WC (in cm) divided by hip circumference (in cm). Systolic blood pressure (SBP) and diastolic blood pressure (DBP) were recorded using a mercury sphygmomanometer for three times at 3–5 minutes intervals.

In CHNS 2009, blood samples were collected from participants after at least 8 hours of overnight fasting. Glucose and haemoglobin A1c (HbA1c) were immediately tested at the survey sites. Then the plasma and serum samples were frozen at −86 °C for later laboratory analyses. Ultimately, all blood samples were analyzed at a national central lab in Beijing.

### Definitions

#### NWCO

Conforming to the Working Group on Obesity in China (WGOC) criteria, a BMI of less than 18.5 kg/m^2^ was defined as underweight, of 18.5 kg/m^2^ to 23.9 kg/m^2^ as normal weight, of 23.9 kg/m^2^ to 27.9 kg/m^2^ as overweight and of 28.0 kg/m^2^ or higher as obesity^33^. Central obesity was defined as a WC > 85 cm in males or >80 cm in females, or a WHtR ≥ 0.5, or a WHR ≥ 0.9 in males or ≥0.85 in females^31–33^. In this study, NWCO was therefore defined as the combinations of normal weight (18.5 kg/m^2^ ≤ BMI < 24.0 kg/m^2^) and 1) a WC > 85 cm in males or >80 cm in females (NWCO by WC); 2) a WHtR ≥ 0.5 (NWCO by WHtR); and 3) a WHR ≥ 0.9 in males or ≥0.85 in females (NWCO by WHR).

#### Demographic, socioeconomic, geographic and behavioural factors

Educational attainments were categorized as no formal education, primary education, middle education (degree from middle school or high school) and higher education (degree from technical or vocational school, university or college, or master’s degree or higher). Since per capita household income (PCHI) was not asymmetrically distributed, we used the terciles of the natural logarithm of PCHI to classify the economic status as poor, middle and rich^45,46^. This was done for different survey years, urban and rural settings separately. All participants were classified into two geographic regions-North China (including Beijing, Liaoning, Heilongjiang, Shandong and Henan in CHNS) and South China (including Shanghai, Jiangsu, Hubei, Hunan, Guangxi, Guizhou and Chongqing in CHNS), according to their residence. According to smoking behaviours, participants were categorized as non-smokers and smokers (including former smokers and current smokers). Similarly, participants were classified as non-drinkers and drinkers (including former drinkers and current drinkers) according to their alcohol drinking behaviours.

#### Cardiometabolic factors

According to the latest guideline on high blood pressure in adults, HP was defined as an SBP ≥ 130 mmHg or a DBP ≥ 90 mmHg, or currently on antihypertensive treatment^47^. Diabetes was defined as a fasting blood glucose ≥126 mg/dL (7.0 mmol/L) or HbA1c ≥ 6.5%, or currently on antidiabetic medication^44^. The homeostasis model of assessment (HOMA) was used to assess IR, IIS, and DIS, where IS was defined as in the upper quartile of the natural logarithm of HOMA_IR, IIS as the lower quartile of the natural logarithm of HOMA-%β, and IS as the lower quartile of the natural logarithm of HOMA-%S^48,49^. In accordance with the Chinese guidelines on prevention and treatment of dyslipidemia in adults, elevated TC was defined as a TC level ≥ 5.18 mmol/L (200 mg/dL), elevated LDL as an LDL level ≥ 3.37 mmol/L (130 mg/dL), low HDL as an HDL level ≤ 1.04 mmol/l (40 mg/dl) and elevated TG as a TG level ≥ 1.70 mmol/l (150 mg/dl)^50^. Inflammation was represented by a high-sensitivity C-reactive protein (hs-CRP) value ≥ 3 mg/dL^44^.

### Statistical analysis

Descriptive analyses were carried out using means with standard deviations (SDs) for continuous variables, and proportions with 95% confidence intervals (CIs) for categorical variables. The prevalence of NWCO in each CHNS wave was standardized to the age distribution of the China Census population in 2010 by the direct method^51^. The generalized estimating equation (GEE) method was used to account for repeated measurements in the same participants across the seven survey rounds from 1993 to 2011, and the robust estimate for the standard errors was used^52,53^. To assess time trends in the prevalence of NWCO across surveys from 1993 to 2011, individual calendar year was included as a single continuous variable and adjusted for age in multivariable logistic regression GEE models. This was separately conducted for various demographic (age, sex), socioeconomic (marital status, education, economic status), geographic (setting, region) and behavioural (smoking, drinking) groups. Similarly, univariable and multivariable logistic regression GEE models were performed by including demographic, socioeconomic, geographic and behavioural variables to investigate the potential demographic, socioeconomic, geographic and behavioural predictors of NWCO.

To assess the cardiometabolic risk of NWCO, a multivariable logistic regression was adopted using the cross-sectional CHNS 2009 data. The comparison was made between the NWCO group and the control group (people with normal weight but without central obesity). The results were reported as odds ratios (ORs) and 95% CI. A p value of less than 0.05 was considered statistically significant and all data analyses were performed using Stata statistical software (version 14.0; Stata Corporation, College Station, TX, USA).

## Supplementary information

Supplementary information
