# Supplementary material for: Temporal trends in normal weight central obesity and its associations with cardiometabolic risk among Chinese adults
Source: Sci Rep. 2019 Apr 1;9:5411. doi: 10.1038/s41598-019-41986-5 (PMC6443661; doi:10.1038/s41598-019-41986-5)
Supplement: Supplementary file 1 — Supplementary information [file 41598_2019_41986_MOESM1_ESM.docx]

**Title:** Temporal trends in normal weight central obesity and its associations with cardiometabolic risk among Chinese adults

Peige Song ^1,2^, Xue Li ^1,2^, Yongjun Bu ^1^, Shibin Ding ^1^, Desheng Zhai ^1^, Erhui Wang ^1^, Zengli Yu ^1^

^1^ School of Public Health, Xinxiang Medical University, Xinxiang, China

^2^ Centre for Global Health Research, University of Edinburgh, Edinburgh, United Kingdom


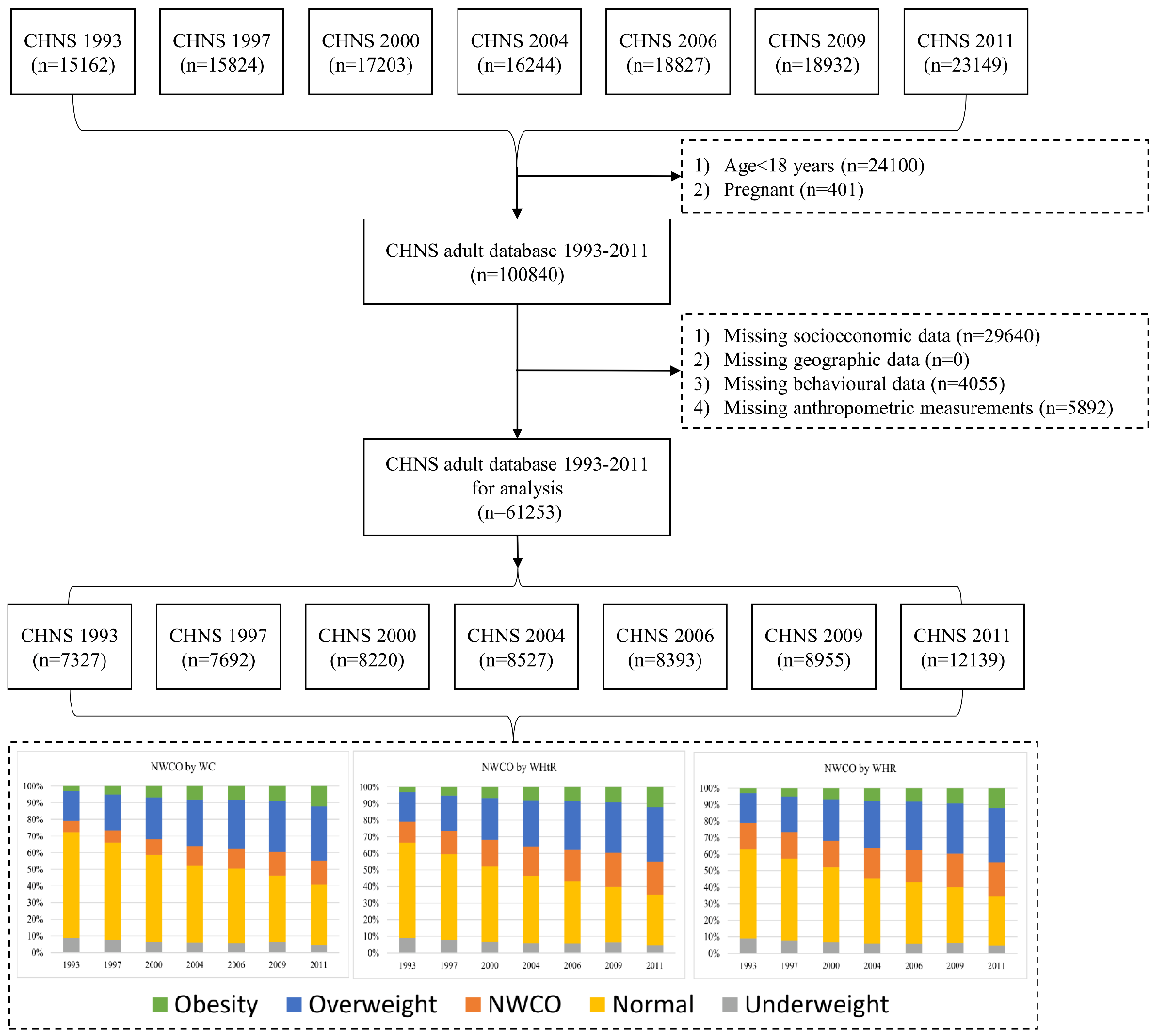


**Figure S1**. Flowchart for selecting records included in the analysis of NWCO prevalence

**Note: the exclusion criteria were in an order as listed in the box, some subjects might meet several exclusion criteria simultaneously; the “normal” group refers to people with normal weight and without central obesity.*

**Table S1**. Comparison of demographic characteristics between the included and excluded records in CHNS 1993-2011

| **Characteristic** | **1993-2011 combined** | | | **1993** | | | **1997** | | | **2000** | | |
| --- | --- | --- | --- | --- | --- | --- | --- | --- | --- | --- | --- | --- |
|  | **Excluded (n=39587)** | **Included (n=61253)** | **p value** | **Excluded (n=3455)** | **Included (n=7327)** | **p value** | **Excluded (n=4151)** | **Included (n=7692)** | **p value** | **Excluded (n=5062)** | **Included (n=8220)** | **p value** |
| **Age group** |  |  | **<0.001** |  |  | **<0.001** |  |  | **<0.001** |  |  | **<0.001** |
| 18-29 years | 41.71 | 14.72 |  | 49.03 | 25.90 |  | 45.87 | 22.57 |  | 44.49 | 17.75 |  |
| 30-39 years | 25.18 | 18.98 |  | 16.32 | 23.90 |  | 16.69 | 21.75 |  | 18.45 | 23.35 |  |
| 40-49 years | 13.68 | 22.94 |  | 11.00 | 21.18 |  | 12.6 | 23.5 |  | 12.15 | 24.06 |  |
| 50-59 years | 7.05 | 20.43 |  | 7.90 | 13.66 |  | 8.36 | 15.00 |  | 8.44 | 16.97 |  |
| 60-69 years | 5.06 | 13.98 |  | 7.41 | 10.47 |  | 7.47 | 11.10 |  | 7.55 | 11.14 |  |
| ≥70 years | 7.31 | 8.94 |  | 8.34 | 4.89 |  | 9.01 | 6.07 |  | 8.93 | 6.73 |  |
| **Sex** |  |  | **0.091** |  |  | **0.005** |  |  | **0.349** |  |  | **0.361** |
| Male | 48.45 | 47.90 |  | 51.00 | 48.12 |  | 48.59 | 49.49 |  | 49.53 | 48.71 |  |
| Female | 51.55 | 52.10 |  | 49.00 | 51.88 |  | 51.41 | 50.51 |  | 50.47 | 51.29 |  |
| **Characteristic** | **2004** | | | **2006** | | | **2009** | | | **2011** | | |
|  | **Excluded (n=4605)** | **Included (n=8527)** | **p value** | **Excluded (n=7497)** | **Included (n=8393)** | **p value** | **Excluded (n=39587)** | **Included (n=61253)** | **p value** | **Excluded (n=39587)** | **Included (n=61253)** | **p value** |
| **Age group** |  |  | **<0.001** |  |  | **<0.001** |  |  | **<0.001** |  |  | **<0.001** |
| 18-29 years | 48.12 | 12.43 |  | 39.82 | 9.92 |  | 37.55 | 9.82 |  | 36.15 | 9.48 |  |
| 30-39 years | 18.85 | 19.46 |  | 31.83 | 18.04 |  | 30.91 | 15.78 |  | 30.15 | 13.99 |  |
| 40-49 years | 11.49 | 22.89 |  | 11.30 | 22.92 |  | 14.86 | 22.78 |  | 19.15 | 23.07 |  |
| 50-59 years | 8.53 | 22.25 |  | 6.68 | 23.98 |  | 6.13 | 24.00 |  | 5.37 | 23.95 |  |
| 60-69 years | 4.99 | 13.59 |  | 3.95 | 14.80 |  | 3.69 | 16.10 |  | 3.48 | 18.01 |  |
| ≥70 years | 8.01 | 9.38 |  | 6.43 | 10.33 |  | 6.87 | 11.52 |  | 5.70 | 11.51 |  |
| **Sex** |  |  | **<0.001** |  |  | **0.388** |  |  | **0.056** |  |  | **0.862** |
| Male | 51.94 | 47.67 |  | 47.71 | 47.03 |  | 46.14 | 47.64 |  | 47.30 | 47.17 |  |
| Female | 48.06 | 52.33 |  | 52.29 | 52.97 |  | 53.86 | 52.36 |  | 52.70 | 52.83 |  |

**Note: values were proportions; Comparisons between the included and excluded subjects were by χ^2^ test.*

**Table S2**. Basic characteristics of all included records in CHNS 1993-2011

| **Characteristic** | **1993-2011 combined (n=61253)** | **1993 (n=7327)** | **1997 (n=7692)** | **2000 (n=8220)** | **2004 (n=8527)** | **2006 (n=8393)** | **2009 (n=8955)** | **2011 (n=12139)** |
| --- | --- | --- | --- | --- | --- | --- | --- | --- |
| **Mean age (year)** | 47.61±15.60 | 42.02±15.43 | 43.51±15.51 | 44.88±15.09 | 48.26±15.23 | 49.56±15.03 | 50.6±15.22 | 51.43±15.11 |
| **Age group** |  |  |  |  |  |  |  |  |
| 18-29 years | 9016 | 1898 | 1736 | 1459 | 1060 | 833 | 879 | 1151 |
|  | (14.72) | (25.90) | (22.57) | (17.75) | (12.43) | (9.92) | (9.82) | (9.48) |
| 30-39 years | 11627 | 1751 | 1673 | 1919 | 1659 | 1514 | 1413 | 1698 |
|  | (18.98) | (23.90) | (21.75) | (23.35) | (19.46) | (18.04) | (15.78) | (13.99) |
| 40-49 years | 14054 | 1552 | 1808 | 1978 | 1952 | 1924 | 2040 | 2800 |
|  | (22.94) | (21.18) | (23.50) | (24.06) | (22.89) | (22.92) | (22.78) | (23.07) |
| 50-59 years | 12516 | 1001 | 1154 | 1395 | 1897 | 2013 | 2149 | 2907 |
|  | (20.43) | (13.66) | (15.00) | (16.97) | (22.25) | (23.98) | (24.00) | (23.95) |
| 60-69 years | 8566 | 767 | 854 | 916 | 1159 | 1242 | 1442 | 2186 |
|  | (13.98) | (10.47) | (11.10) | (11.14) | (13.59) | (14.80) | (16.10) | (18.01) |
| ≥70 years | 5474 | 358 | 467 | 553 | 800 | 867 | 1032 | 1397 |
|  | (8.94) | (4.89) | (6.07) | (6.73) | (9.38) | (10.33) | (11.52) | (11.51) |
| **Sex** |  |  |  |  |  |  |  |  |
| Male | 29341 | 3526 | 3807 | 4004 | 4065 | 3947 | 4266 | 5726 |
|  | (47.90) | (48.12) | (49.49) | (48.71) | (47.67) | (47.03) | (47.64) | (47.17) |
| Female | 31912 | 3801 | 3885 | 4216 | 4462 | 4446 | 4689 | 6413 |
|  | (52.10) | (51.88) | (50.51) | (51.29) | (52.33) | (52.97) | (52.36) | (52.83) |
| **Marital status** |  |  |  |  |  |  |  |  |
| Single | 10752 | 1565 | 1573 | 1551 | 1473 | 1316 | 1412 | 1862 |
|  | (17.55) | (21.36) | (20.45) | (18.87) | (17.27) | (15.68) | (15.77) | (15.34) |
| Married | 50501 | 5762 | 6119 | 6669 | 7054 | 7077 | 7543 | 10277 |
|  | (82.45) | (78.64) | (79.55) | (81.13) | (82.73) | (84.32) | (84.23) | (84.66) |
| **Education** |  |  |  |  |  |  |  |  |
| No formal education | 14782 | 2384 | 2213 | 1867 | 1794 | 2076 | 2047 | 2401 |
|  | (24.13) | (32.54) | (28.77) | (22.71) | (21.04) | (24.73) | (22.86) | (19.78) |
| Primary education | 12372 | 1577 | 1718 | 1846 | 1983 | 1525 | 1734 | 1989 |
|  | (20.20) | (21.52) | (22.33) | (22.46) | (23.26) | (18.17) | (19.36) | (16.39) |
| Middle education | 26950 | 3028 | 3244 | 3737 | 3839 | 3719 | 4041 | 5342 |
|  | (44.00) | (41.33) | (42.17) | (45.46) | (45.02) | (44.31) | (45.13) | (44.01) |
| Higher education | 7149 | 338 | 517 | 770 | 911 | 1073 | 1133 | 2407 |
|  | (11.67) | (4.61) | (6.72) | (9.37) | (10.68) | (12.78) | (12.65) | (19.83) |
| **Economic status** |  |  |  |  |  |  |  |  |
| Poor | 18026 | 2263 | 2435 | 2578 | 2560 | 2370 | 2593 | 3227 |
|  | (29.43) | (30.89) | (31.66) | (31.36) | (30.02) | (28.24) | (28.96) | (26.58) |
| Middle | 20370 | 2437 | 2548 | 2700 | 2831 | 2794 | 2962 | 4098 |
|  | (33.26) | (33.26) | (33.13) | (32.85) | (33.20) | (33.29) | (33.08) | (33.76) |
| Rich | 22857 | 2627 | 2709 | 2942 | 3136 | 3229 | 3400 | 4814 |
|  | (37.32) | (35.85) | (35.22) | (35.79) | (36.78) | (38.47) | (37.97) | (39.66) |
| **Setting** |  |  |  |  |  |  |  |  |
| Rural | 40673 | 4985 | 5243 | 5600 | 5890 | 5791 | 6172 | 6992 |
|  | (66.40) | (68.04) | (68.16) | (68.13) | (69.07) | (69.00) | (68.92) | (57.60) |
| Urban | 20580 | 2342 | 2449 | 2620 | 2637 | 2602 | 2783 | 5147 |
|  | (33.60) | (31.96) | (31.84) | (31.87) | (30.93) | (31.00) | (31.08) | (42.40) |
| **Region** |  |  |  |  |  |  |  |  |
| North | 24822 | 2532 | 2775 | 3416 | 3674 | 3643 | 3911 | 4871 |
|  | (40.52) | (34.56) | (36.08) | (41.56) | (43.09) | (43.41) | (43.67) | (40.13) |
| South | 36431 | 4795 | 4917 | 4804 | 4853 | 4750 | 5044 | 7268 |
|  | (59.48) | (65.44) | (63.92) | (58.44) | (56.91) | (56.59) | (56.33) | (59.87) |
| **Smoking** |  |  |  |  |  |  |  |  |
| Non-smoker | 41611 | 4820 | 5162 | 5596 | 5743 | 5750 | 6135 | 8405 |
|  | (67.93) | (65.78) | (67.11) | (68.08) | (67.35) | (68.51) | (68.51) | (69.24) |
| Smoker | 19642 | 2507 | 2530 | 2624 | 2784 | 2643 | 2820 | 3734 |
|  | (32.07) | (34.22) | (32.89) | (31.92) | (32.65) | (31.49) | (31.49) | (30.76) |
| **Alcohol drinking** |  |  |  |  |  |  |  |  |
| Non-drinker | 40169 | 4661 | 4858 | 5278 | 5709 | 5695 | 5970 | 7998 |
|  | (65.58) | (63.61) | (63.16) | (64.21) | (66.95) | (67.85) | (66.67) | (65.89) |
| Drinker | 21084 | 2666 | 2834 | 2942 | 2818 | 2698 | 2985 | 4141 |
|  | (34.42) | (36.39) | (36.84) | (35.79) | (33.05) | (32.15) | (33.33) | (34.11) |
| **Mean BMI (kg/m^2^)** | 23.01±3.63 | 21.86±2.83 | 22.32±3.09 | 22.76±3.22 | 23.06±3.35 | 23.18±3.53 | 23.30±3.43 | 23.94±4.62 |
| **Mean WC (cm)** | 80.56±10.33 | 75.76±8.89 | 77.56±9.39 | 79.38±9.74 | 80.85±9.86 | 81.35±9.91 | 82.59±10.24 | 83.89±11.04 |
| **Mean WHtR** | 0.50±0.06 | 0.47±0.06 | 0.48±0.06 | 0.49±0.06 | 0.50±0.06 | 0.51±0.06 | 0.51±0.06 | 0.52±0.07 |
| **Mean WHR** | 0.86±0.09 | 0.84±0.07 | 0.85±0.07 | 0.85±0.07 | 0.87±0.11 | 0.87±0.07 | 0.87±0.08 | 0.88±0.13 |

**Note: values were n (%) and mean±SD; BMI=body mass index; WC=waist circumference; WHtR=waist to height ratio; WHR=waist to hip ratio.*

**Table S3**. Basic characteristics of the records of normal weight people in CHNS 2009

| **Characteristic** | **Subjects with normal weight (n=4244)** |
| --- | --- |
| **Mean age (year)** | 50.20±15.43 |
| **Age group** |  |
| 18-29 years | 457 (10.77) |
| 30-39 years | 678 (15.98) |
| 40-49 years | 960 (22.62) |
| 50-59 years | 1002 (23.61) |
| 60-69 years | 659 (15.53) |
| ≥70 years | 488 (11.5) |
| **Sex** |  |
| Male | 1998 (47.08) |
| Female | 2246 (52.92) |
| **Marital status** |  |
| Single | 659 (15.53) |
| Married | 3583 (84.47) |
| **Education** |  |
| No formal education | 992 (23.37) |
| Primary education | 823 (19.39) |
| Middle education | 1919 (45.22) |
| Higher education | 510 (12.02) |
| **Economic status** |  |
| Poor | 1263 (29.76) |
| Middle | 1454 (34.26) |
| Rich | 1527 (35.98) |
| **Setting** |  |
| Rural | 3012 (70.97) |
| Urban | 1232 (29.03) |
| **Region** |  |
| North | 1604 (37.79) |
| South | 2640 (62.21) |
| **Smoking** |  |
| Non-smoker | 2861 (67.41) |
| Smoker | 1383 (32.59) |
| **Alcohol drinking** |  |
| Non-drinker | 2871 (67.65) |
| Drinker | 1373 (32.35) |
| **Mean BMI(kg/m^2^)** | 21.52±1.48 |
| **Mean WC (cm)** | 78.38±7.43 |
| **Mean WHtR** | 0.49±0.05 |
| **Mean WHR** | 0.86±0.07 |

**Note: values were n (%) and mean±SD; BMI=body mass index; WC=waist circumference; WHtR=waist to height ratio; WHR=waist to hip ratio.*

**Table S4**. Unadjusted odds ratios of demographic, socioeconomic and behavioural factors of normal weight central obesity in univariable logistic regressions, CHNS 1993-2011

| **Characteristic** | **NWCO by WC** | | **NWCO by WHtR** | | **NWCO by WHR** | | |
| --- | --- | --- | --- | --- | --- | --- | --- |
|  | **Unadjusted odds ratio (95% CI)** | **p value** | **Unadjusted odds ratio (95% CI)** | **p value** | **Unadjusted odds ratio (95% CI)** | **p value** |  |
| **Survey year** |  | **<0.001** |  | **<0.001** |  | **<0.001** |  |
| 1993 | 1.00 (reference) |  | 1.00 (reference) |  | 1.00 (reference) |  |  |
| 1997 | 1.17 (1.03-1.32) | 0.012 | 1.14 (1.04-1.25) | 0.005 | 1.07 (0.99-1.17) | 0.099 |  |
| 2000 | 1.51 (1.35-1.70) | <0.001 | 1.37 (1.26-1.50) | <0.001 | 1.08 (0.99-1.17) | 0.074 |  |
| 2004 | 1.91 (1.70-2.13) | <0.001 | 1.53 (1.40-1.68) | <0.001 | 1.26 (1.16-1.37) | <0.001 |  |
| 2006 | 2.05 (1.83-2.29) | <0.001 | 1.69 (1.54-1.84) | <0.001 | 1.37 (1.26-1.48) | <0.001 |  |
| 2009 | 2.40 (2.15-2.68) | <0.001 | 1.86 (1.71-2.03) | <0.001 | 1.42 (1.31-1.53) | <0.001 |  |
| 2011 | 2.54 (2.28-2.82) | <0.001 | 1.84 (1.70-2.00) | <0.001 | 1.44 (1.33-1.55) | <0.001 |  |
| **Age group** |  | **<0.001** |  | **<0.001** |  | **<0.001** |  |
| 18-29 years | 1.00 (reference) |  | 1.00 (reference) |  | 1.00 (reference) |  |  |
| 30-39 years | 1.39 (1.24-1.56) | <0.001 | 1.65 (1.49-1.82) | <0.001 | 1.11 (1.03-1.21) | 0.009 |  |
| 40-49 years | 1.94 (1.74-2.16) | <0.001 | 2.41 (2.19-2.65) | <0.001 | 1.32 (1.22-1.43) | <0.001 |  |
| 50-59 years | 2.38 (2.13-2.65) | <0.001 | 3.16 (2.87-3.47) | <0.001 | 1.60 (1.48-1.74) | <0.001 |  |
| 60-69 years | 2.82 (2.52-3.16) | <0.001 | 3.99 (3.62-4.40) | <0.001 | 1.84 (1.69-2.00) | <0.001 |  |
| ≥70 years | 3.77 (3.33-4.25) | <0.001 | 5.46 (4.91-6.08) | <0.001 | 2.60 (2.37-2.86) | <0.001 |  |
| **Sex** |  |  |  |  |  |  |  |
| Male | 1.00 (reference) |  | 1.00 (reference) |  | 1.00 (reference) |  |  |
| Female | 1.30 (1.22-1.38) | <0.001 | 1.46 (1.39-1.54) | <0.001 | 1.64 (1.56-1.73) | <0.001 |  |
| **Marital status** |  |  |  |  |  |  |  |
| Single | 1.00 (reference) |  | 1.00 (reference) |  | 1.00 (reference) |  |  |
| Married | 1.15 (1.06-1.24) | <0.001 | 1.12 (1.05-1.20) | 0.001 | 0.99 (0.93-1.05) | 0.625 |  |
| **Education** |  | **<0.001** |  | **<0.001** |  | **<0.001** |  |
| No formal education | 1.00 (reference) |  | 1.00 (reference) |  | 1.00 (reference) |  |  |
| Primary education | 0.76 (0.70-0.83) | <0.001 | 0.67 (0.63-0.72) | <0.001 | 0.70 (0.65-0.75) | <0.001 |  |
| Middle education | 0.70 (0.65-0.76) | <0.001 | 0.51 (0.48-0.54) | <0.001 | 0.59 (0.56-0.63) | <0.001 |  |
| Higher education | 0.81 (0.73-0.90) | <0.001 | 0.47 (0.43-0.52) | <0.001 | 0.61 (0.56-0.66) | <0.001 |  |
| **Economic status** |  | **0.850** |  | **<0.001** |  | **<0.001** |  |
| Poor | 1.00 (reference) |  | 1.00 (reference) |  | 1.00 (reference) |  |  |
| Middle | 0.95 (0.89-1.02) | 0.139 | 0.90 (0.86-0.95) | <0.001 | 0.91 (0.87-0.96) | <0.001 |  |
| Rich | 0.99 (0.93-1.06) | 0.784 | 0.89 (0.84-0.94) | <0.001 | 0.89 (0.85-0.94) | <0.001 |  |
| **Setting** |  |  |  |  |  |  |  |
| Rural | 1.00 (reference) |  | 1.00 (reference) |  | 1.00 (reference) |  |  |
| Urban | 1.27 (1.19-1.35) | <0.001 | 0.97 (0.92-1.03) | 0.327 | 0.93 (0.88-0.98) | 0.007 |  |
| **Region** |  |  |  |  |  |  |  |
| North | 1.00 (reference) |  | 1.00 (reference) |  | 1.00 (reference) |  |  |
| South | 0.84 (0.79-0.90) | <0.001 | 1.21 (1.14-1.28) | <0.001 | 1.22 (1.15-1.28) | <0.001 |  |
| **Smoking** |  |  |  |  |  |  |  |
| Non-smoker | 1.00 (reference) |  | 1.00 (reference) |  | 1.00 (reference) |  |  |
| Smoker | 0.89 (0.83-0.95) | <0.001 | 0.87 (0.82-0.91) | <0.001 | 0.80 (0.76-0.85) | <0.001 |  |
| **Alcohol drinking** |  |  |  |  |  |  |  |
| Non-drinker | 1.00 (reference) |  | 1.00 (reference) |  | 1.00 (reference) |  |  |
| Drinker | 0.89 (0.84-0.95) | <0.001 | 0.83 (0.79-0.88) | <0.001 | 0.81 (0.77-0.85) | <0.001 |  |

**Note:* *values were odds ratios (95% confidence interval); NWCO=normal weight central obesity;* *WC=waist circumference; WHtR=waist to height ratio; WHR=waist to hip ratio; Comparisons were between the NWCO group and the control group (without NWCO).*
